# The orosomucoid 1 protein (α1 acid glycoprotein) is overexpressed in odontogenic myxoma

**DOI:** 10.1186/1477-5956-10-49

**Published:** 2012-08-13

**Authors:** Alejandro García-Muñoz, Mario A Rodríguez, Ronell Bologna-Molina, Febe E Cázares-Raga, Fidel C Hernández-Hernández, J Eduardo Farfán-Morales, Juan J Trujillo, Carlos Licéaga-Escalera, Guillermo Mendoza-Hernández

**Affiliations:** 1 Departamento de Infectómica y Patogénesis Molecular, CINVESTAV-IPN, México, D.F., México; 2 Departamento de Investigación, Escuela de Odontología, Universidad Juárez del Estado de Durango, Durango, México; 3 Facultad de Odontología, Universidad de la República (UDELAR), Montevideo, Uruguay; 4 Laboratorio de Patología Molecular, Instituto Nacional de Pediatría, México, D.F., México; 5 Departamento de Cirugía Maxilofacial, Hospital Juárez de México, México, D.F., México; 6 Departamento de Bioquímica, Facultad de Medicina, UNAM, México, D.F., México

**Keywords:** Odontogenic myxoma, Dental follicle, Proteomic analysis, Orosomucoid 1, α1 acid glycoprotein

## Abstract

**Background:**

Odontogenic myxoma (OM) is a benign, but locally invasive, neoplasm occurring in the jaws. However, the molecules implicated in its development are unknown. OM as well as Dental Follicle (DF), an odontogenic tissue surrounding the enamel organ, is derived from ectomesenchymal/mesencyhmal elements. To identify some protein that could participate in the development of this neoplasm, total proteins from OM were separated by two-dimensional electrophoresis and the profiles were compared with those obtained from DF, used as a control.

**Results:**

We identified eight proteins with differential expression; two of them were downregulated and six upregulated in OM. A spot consistently overexpressed in odontogenic myxoma, with a molecular weight of 44-kDa and a *pI* of 3.5 was identified as the orosomucoid 1 protein. Western blot experiments confirmed the overexpression of this protein in odontogenic myxoma and immunohistochemical assays showed that this protein was mainly located in the cytoplasm of stellate and spindle-shaped cells of this neoplasm.

**Conclusion:**

Orosomucoid 1, which belongs to a group of acute-phase proteins, may play a role in the modulation of the immune system and possibly it influences the development of OM.

## Background

Odontogenic Myxoma (OM) is a relatively rare, benign neoplasm occurring in the jaws. This neoplasm is characterized by the presence of stellate and spindle-shaped cells embedded in an abundant myxoid or mucoid extracellular matrix. OM represents 3-20% of all odontogenic tumours and, in most studies, OM is the third most frequent odontogenic tumor [1]. Conservative surgery by enucleation and curettage is recommended when lesions of OM are smaller than 3 cm, but a segmental resection with immediate reconstruction is preferred in patients affected by bigger tumors [2].

OM as well as Dental Follicle (DF), an odontogenic tissue surrounding the enamel organ, and the dental papilla of the developing tooth germ prior to eruption [3], is derived from ectomesenchymal/mesencyhmal elements. Thus, OM could be mimicked by DF and dental papilla, both containing myxoid areas [4-6]. Indeed, a pathologist who is not familiar with the histology of a tooth germ can mistake a myxoid DF for an OM [6].

Up to now there are few studies comparing molecules of OM with other odontogenic mesenchymal tissues. Some authors compared the expression of α-SMA, S-100 and vimentin between OM and other mesenchymal tissues [7,8], but not substantial differences were found. Another study described that the hyaluronic acid concentration in OM is four times higher than that of other glycosaminoglycans, such as chondroitin sulphate, which is inversely found in mesenchymal tissues from dental pulp, gingival and periodontal ligament, but not in DF [9]. It was also reported that 90% of OM cells expressed the metalloproteinase 2 (MMP-2), while only 10% of the cells in DF and myxoid dental pulp expressed this protein [10]. These authors also showed an increased expression of Bcl-2 and Bcl-x in OM. However, other studies reported less than 1% of Bcl-2 positive cells in OM [11,12]. Finally, some works have been focused on the histological changes that occur in the hyperplastic DF and normal DF of impacted third molars and their histological association to OM and the possibilities of misinterpretation as OM [4,6,13,14].

In recent years, the use of high-throughput genomics and proteomics has expanded rapidly in biomedical science. These technologies have evolved and make possible several discoveries in clinical cancer research, including the identification of biomarkers, molecular classification of tumors, molecular prediction of metastasis, treatment response, and prognosis. Particularly, the study of the proteome, the collection of all the proteins expressed from specific cells in all isoforms, polymorphisms and post-translational modifications [15], has allowed the detection of new biomarkers in diverse types of neoplasitic tissues, for example in urinary bladder cancer [16], ovarian carcinomas [17], oral squamous cell carcinoma [18], and lung cancer [19]. However, in the literature we have not found any previous study about OM or other odontogenic tumors using this approach.

To identify some proteins that could participate in the biological behavior of OM, in this work we used the proteomic technology based on 2-dimensional electrophoresis (2DE) combined with liquid chromatography-tandem mass spectrometry (LC-MS/MS) for comparing odontogenic myxoma (neoplastic) versus dental follicle (normal) tissues. A spot consistently overexpressed in odontogenic myxoma was identified as the orosomucoid 1 protein, which was located in the cytoplasm of the tumor cells.

## Results

### Protein profiles of OM and DF

**Figure 1:**
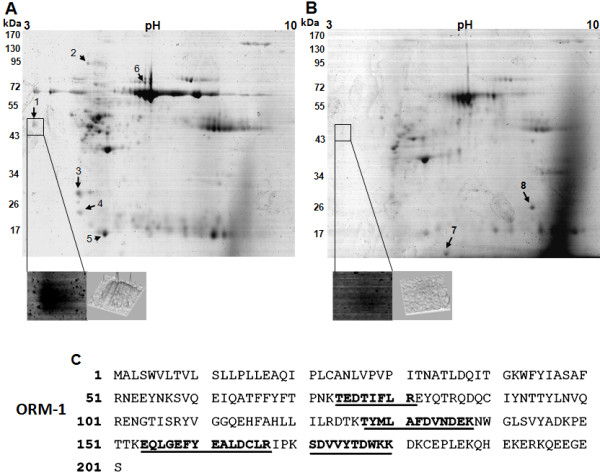
**2DE protein profiles of Odontogenic Myxoma (OM) and Dental Follicle (DF) and identification of ORM1.** Proteins from DF and OM were extracted and separated in 2DE. Then, gels were stained with Colloidal Coomassie Blue G-250. (**A**) Protein profile of OM. (**B**) Protein profile of DF. Proteins differentially expressed between both samples are numbered and one of the spots that consistently showed significant upregulation in OM is indicated by a frame. Under each gel are shown the magnifications and differential intensity analyses for the spot indicated by the frame. (**C**) This spot was excised from gels and subsequently analyzed by LC-MS/MS. This analysis identified this spot as the orosomucoid 1 protein (ORM1), which amino acid sequence is shown. The peptides identified by LC-MS/MS are underlined.

To identify proteins with differential expression in OM with respect to DF, the protein extracts from five OM (Table1) and five DF samples were analyzed by 2-DE using a wide range ampholyte pH 3–10 and profiles were visualized by colloidal Coomassie Blue G-250 staining. To minimize gel to gel variation, two-dimensional gels for each sample were realized at least twice. Both, DF and OM samples showed similar protein profiles, including more than 100 spots with molecular masses ranging from >170 to 5 kDa and pI values between 3 and 10 (Figure1A, B). Several protein spots consistently displayed significant differences in expression between OM and DF. Figure1 shows representative 2-DE of OM and DF with spots subjected to mass spectrometry and their identification numbers; the identified spots are listed in Table2.

**Table 1:** Details of cases

**Age**	**Sex**	**Location**	**Approximate evolution**	**Approximate diameter**	**Symptoms**
35	Female	Jaw/posterior	8 Months	3 × 2 cm/with expanded cortical	Increased volume/asymptomatic
25	Female	Jaw/posterior	12 Months	4 × 2 cm/with expanded cortical	Increased volume/paresthesia
16	Female	Maxillary/anterior	3 Months	4 × 5 cm/with expanded cortical	Increased volume/asymptomatic
15	Male	Jaw/posterior	7 Months	9 × 5 cm/with expanded cortical	Increased volume/asymptomatic
25	Female	Jaw/ramus	3-5 years	4 × 5 cm/with expanded cortical	Increased volume/little pain

**Table 2:** Identification of proteins with differential expression in Odontogenic Myxoma

**Spot ID**	**NCBI No.**	**Name**	**Theoretical Protein ID Mass KDa/pl**	**Mascot Score**	**Sequence Coverage**	**Major functions**
Up-regulated proteins						
1	gi|112877	Orosomucoid-1	23497/4.93	182	21%	Acute phase with inflammatory and immunomodulating properties
2	gi|4507677	GRP94	92696/4.76	471	21%	Molecular chaperone and cell signalling
3	gi|4507651	Tropomyosin alpha-4	28619/4.67	383	35%	Calcium binding and acti-binding
4	gi|4507953	14-3-3 protein	27899/4.73	341	40%	Cell signaling, cycle control, apoptosis and metabolism
5	gi|90108664	Apolipoprotein A-1	28061/5.27	717	57%	Lipid transport, metabolism, apoptosis and autophagy
6	gi|15783061	Serum Albumin in a Complex With Myristic Acid And Tri-lodobenzoic Acid	67988/5.69	239	33%	Protein of binding to cations, fatty acids, bilirubin and other
Down-regulated proteins						
7	gi|494066	Glutathione S-transferase	23438/5.44	143	22%	Detoxify endogenous and environmental substances
8	gi|4502517	Carbonic anhydrase 1	28909/6.59	376	55%	Ubiquitous metalloenzyme; bone resorption, calcification, ion transport, acid–base transport and metabolic processes

Accession numbers are from the MASCOT database (http://www.matrixscience.com).

### Identification of orosomucoid-1 (ORM1)

One spot that consistently showed significant upregulation in OM was a molecule of approximately 44 kDa with a pI value around of 3.5 (Figure1, square). The results of the data query from the LC-MS/MS analysis indicated that four mass values of this spot matched with a human protein called orosomucoid 1 (ORM1), or alpha 1 acid glycoprotein, with a sequence coverage of 20.3% (Figure1C).

### Expression of ORM1 in Odontogenic Myxoma vs Dental Follicle

**Figure 2:**
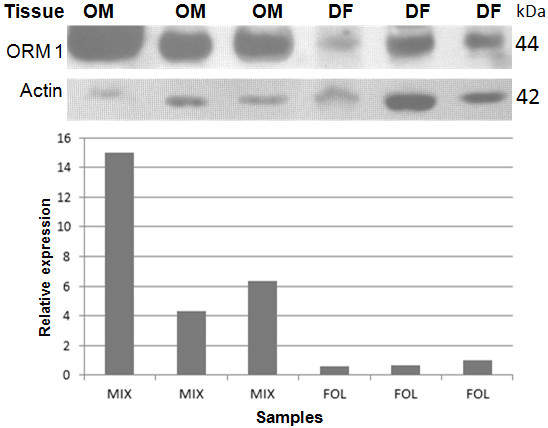
**Western blot assays for detection of the ORM 1 protein.** Protein extracts from OM and DF samples were separated by PAGE-SDS and submitted to Western blot assays using antibodies against ORM1 and against actin, the latter used as an internal control. Relative intensities of the bands recognized by the antibodies were documented and analyzed by densitometry. The relative expression of ORM1 in a DF sample was arbitrary taken as 1.

To verify the differential expression of ORM1, which could play important functional roles in the development of OM, we performed Western blot assays on independent samples of OM and DF with a commercial monoclonal antibody. This analysis showed that the antibody strongly recognized a band of approximately 44 kDa, the expected molecular weight for the ORM1 protein, in all samples of OM analyzed, whereas this band was detected with minor intensity in samples of DF (Figure2). A 42-kDa band with roughly similar intensity was detected in all samples by an anti-actin antibody, used as internal control (Figure2). We performed densitometric analysis of the bands detected by those antibodies and the relative expression of ORM1 in a DF sample was arbitrarily taken as 1. This analysis showed that compared with DF, OM presented from 4.5- to 15-fold increased expression of ORM1 (Figure2). This result confirmed that ORM1 is highly expressed in OM.

### Expression pattern of ORM1 in Odontogenic Myxoma vs normal Dental Follicle

**Figure 3:**
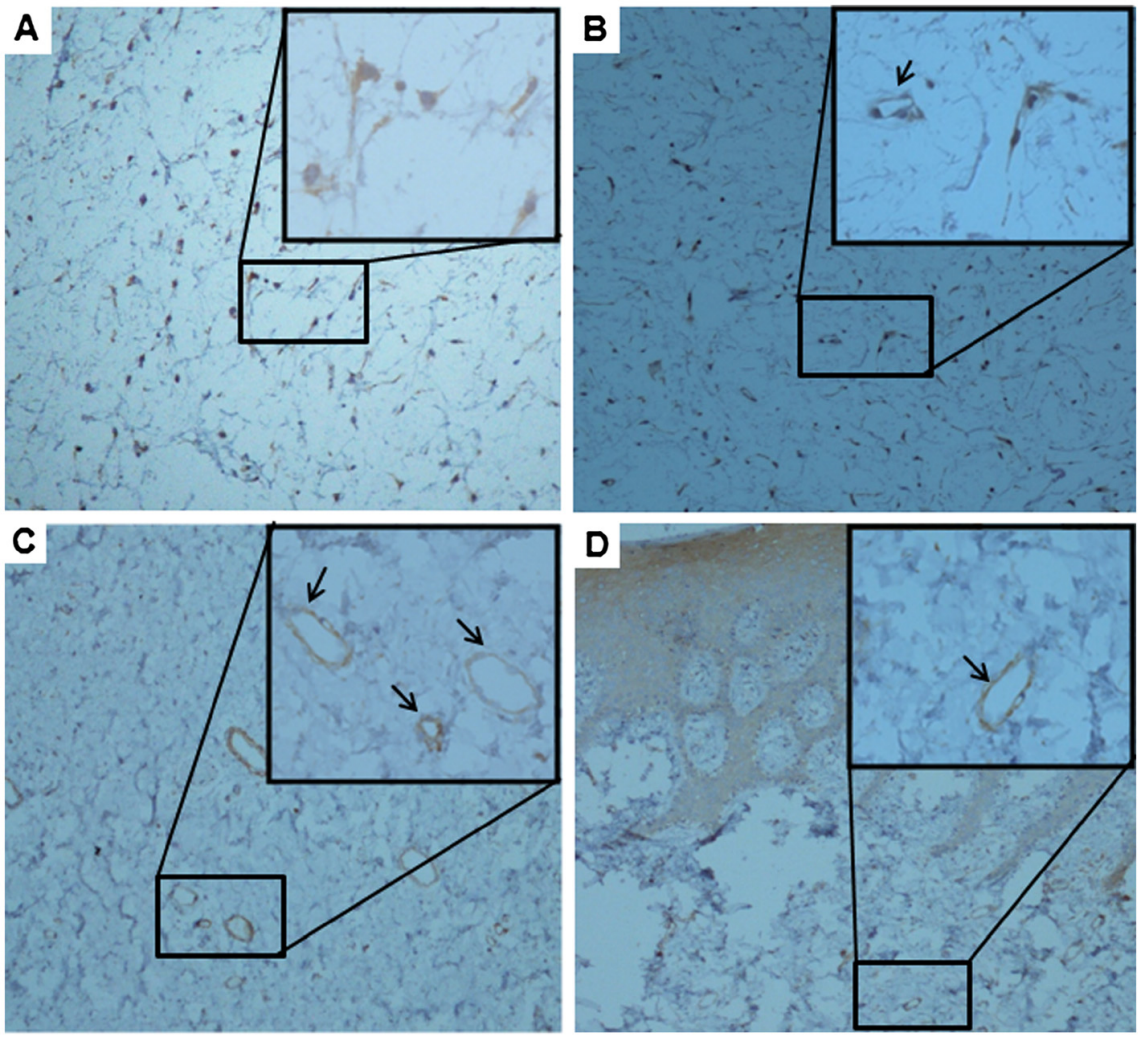
**In situ expression of ORM1 in OM and DF.** Tissue sections from different samples of OM (**A**, **B**) and DF (**C**, **D**) were incubated with a monoclonal antibody against ORM1, then, with a biotinylated antimouse antibody, and finally with the streptavidine/peroxidase complex. The reaction products were visualized by the incubation with 3,3´-diaminobenzidine-H_2_O_2_ substrate. Finally, samples were analyzed by optic microscopy (20X). Insets show magnifications (40X) of the marked areas. Arrows indicate endothelial cells of blood vessels (B-D). Panel D also showed contaminant epithelial cells positive for ORM1.

To determine the in situ expression of ORM1 in the tumoral mass of OM, we performed an immunohistochemical assay on fourteen OM and ten DF samples using the monoclonal antibody against this protein. OM displayed positive cytoplasmic staining in majority of the stellate and spindle-shaped cells in all the analyzed samples (Figure3A, B), while mesenchymal cells of DF did not exhibit immunopositivity (Figure3C, D). In addition, in both tissues (OM and DF) the endothelial cells of large and small blood vessels showed ORM1 positivity (Figure3A-D). Positivity was also found in contaminant epithelial cells of DF (Figure3D).

## Discussion

The pathogenesis and source of OM is still controversial. Some authors have proposed an odontogenic origin, particularly from the dental follicle or from the periodontal ligament [20,21]. Other authors have suggested that OM may be the result of a myxoid change in a pre-existing mesenchymal lesion or that it may represent a degenerative form of odontogenic fibroma [22]. In contrast, some authors have posed that the OM has a myofibroblastic origin [23,24]. Nevertheless, in the last years many studies have compared the biochemical composition, particularly in the extracellular matrix, of OM with organs (dental pulp, dental follicles, gingival tissue and periodontal ligament) of a developing tooth [11,25,26]. The results obtained in the present study showed that the protein profiles of OM and DF are very similar; supporting the notion that OM could originate from DF.

To analyze proteins differentially expressed in OM and DF we used a proteomic approach based on 2-DE and peptide mass fingerprint by LC-MS/MS. This proteomic analysis revealed the variation of eight proteins identified (Table1).

Expression of carbonic anhydrase I (CA I) and glutathione S-transferase (GST) was downregulated in OM. Carbonic anhydrases catalyze the hydration of carbon dioxide and forms bicarbonate. CA I not only enhances the hydration reaction of CO_2_, but it also promotes the combining of bicarbonate with calcium to form the solid precipitant of calcium carbonate [27], a principal component of bones. Although OM is considered a benign neoplasm, it shows a high potential for bone resorption [28]. Thus, downregulation of CA I may affect the balance between bone resorption and apposition. On the other hand, GSTs are a large family of enzymes that catalyze the conjugation of reduced glutathione through a sulfhydryl group to electrophilic sites on a wide variety of substrates that could lead to the generation of reactive oxygen species (ROS) [29]. The products of GST catalysis are more water soluble, promoting ROS detoxification and thereby protecting tissues from oxidative damage. Thus, *GST* could be acting as a caretaker protein by protecting cells against genome damage induced by carcinogens and as a tumor-suppressor protein leading to tumor growth when inactivated [30]. It is, therefore, speculative that downexpression of GST in OM would lead to genome damage accumulation and be further injurious to the oral tissue.

A glucose-regulated protein (GRP94), albumin in a complex with myristic acid and tri-iodobenzoic acid, the tropomyosin alpha-4, the 14-3-3 protein zeta/delta, the apolipoprotein A-I, and the orosomucoid-1 protein were up-regulated in OM. Interestingly, overexpression of tropomyosin alpha-4 was also detected in esophageal squamous cell carcinoma [31], although their participation in tumor development remains to be investigated.

GRPs refer to a set of endoplasmic reticulum (ER) chaperones that have multiple functions in maintaining cellular homeostasis [32]. The endoplasmic reticulum stress pathways and the GRPs have been linked to cancer growth and drug resistance [33]. GRPs represent novel markers for cancer progression and chemo-responsiveness, as well as targets for cancer therapy. GRP94, also known as gp96, is the most abundant glycoprotein in ER and its overexpression associates with cellular transformation, tumorigenicity and decreased sensitivity to X-rays, whereas suppression of GRP94 sensitizes cells to etoposide treatment [32].

The 14-3-3 proteins belong to a family consisting of highly conserved acidic proteins with molecular weights of 25–30 kDa. They participate in phosphorylation-dependent protein-protein interactions that control progression through the cell cycle, initiation and maintenance of DNA damage checkpoints, activation of MAP kinases, prevention of apoptosis and coordination of integrin signaling and cytoskeletal dynamics [34]. Accumulating evidence now supports the concept that either an abnormal state of 14-3-3 protein expression, or dysregulation of 14-3-3/client protein interactions, contributes to the development of a large number of human diseases. In particular, clinical investigations in the field of oncology have demonstrated a correlation between upregulated 14-3-3 levels and poor survival of cancer patients [35].

ApoA-I is the major protein in HDL and plays an important role in reverse cholesterol transport by extracting cholesterol and phospholipids from peripheral cells and transferring it to the liver for excretion. In addition to its antiatherogenic properties, apoA-I also possesses anti-inflammatory and antioxidant properties [36]. Decreased levels of Apolipoprotein were found in a variety of cancer [37-39], but such as in OM, Apolipoprotein A-I was increased in breast cancer and brain metastases in lung cancer [40,41]. This controversy about the regulation of ApoA-I in cancer cells needs to be clarified in future studies.

By proteomics,Western blot and immunohistochemical assays, in the present study we showed that the ORM1 protein is overexpressed in OM. Interestingly, the same strategies allowed the identification of increased levels of ORM1 in urine samples of patients with urinary bladder cancer [16]. Moreover, increased levels of ORM1 have been reported in the sera of patients with different malignant diseases, including squamous cell carcinoma of head and neck [42-46].

ORM1 belongs to a group of acute-phase proteins found in plasma. Such proteins undergo dramatic changes in concentration as a response of the organism to a disturbance of its homeostasis. These plasmatic proteins constitute a group of serum factors related to different immunological regulator functions and they have also been associated with tumor development and growth. However, it is uncertain whether the serum levels of acute-phase proteins, such as ORM1, increase as a response of the host to tumor growth or as a consequence of neoplastic cell production.

Human hepatocytes are normally the site of ORM1 production, but endothelial cells and some tumor cells can also produce it [16,45,47]. Additionally, some studies have shown that ORM1 is synthesized by lymphocytes, granulocytes, macrophages and monocytes [48,49]. In the present study the expression of ORM1 in OM was mainly detected in the cytoplasm of stellate and spindle-shaped cells. However, this protein was also detected in the endothelial cells of blood vessels in both OM and DF tissue samples. It has been reported that ORM1 alone enhances migration but not the proliferation of human dermal microvascular endothelial cells, but in the presence of ORM1 and the vascular endothelial growth factor A (VEGF-A) the endothelial cells are capable to induce the development of endothelial tubes, suggesting that ORM1 seems to be involved in the regulation of angiogenesis [50]. Irmak et al. [16] proposed that the highest increase of ORM1 levels in advanced stages of urinary bladder cancer, which correspond to a vascularized tumor, could be due in part to the production of this protein by the augmented number of endothelial cells of angiogenically active blood vessels. The pro-angiogenic collaborative property of ORM1 may possibly occur in OM, but further studies with the association of angiogenic markers and ORM1 in OM are needed to test this hypothesis.

The presence of ORM1 in odontogenic myxoma also suggests a possible immunomodulatory function and a role in the growth and invasion potential of the tumoral cells. ORM1 is able to inhibit polymorphonuclear neutrophil activation and is considered a natural anti-inflammatory, anti-neutrophil, anti-complement and immunomodulatory agent [51]. Thus, the overexpression of ORM1 in OM may inhibit the immune response, resulting in an increase of tumor cell proliferation. Alternatively, the high expression of ORM1 in OM could represent a defense mechanism against proliferation and invasion of the tumor cells, similar to what occurs in colon cancer cells. In the latter neoplastic cells, the overexpression of ORM1 results in a reduced colony-forming capacity, as well as in a decrease of invasion and adhesion, whereas the inhibition of the expression of ORM1 by antisense oligodeoxynucleotides produces an increase of these events [52]. However, due to the multiple roles that have been described for ORM1 [51], it is difficult at this moment to assign just one specific function of this protein in OM.

On the other hand, ORM1 has very high carbohydrate content (45%). Glycoproteins contain carbohydrate residues from less than 1% until 80% of their total molecular weight and when glycoproteins include more than 4% of carbohydrates they are often called mucoproteins, because they have a high viscosity [53]. Macroscopically OM is an infiltrative mass of mucoid or slimy material, with a high viscosity. It is a slow growing tumor consisting of an accumulation of mucoid ground substance and, in some instances this mucoid mass can be infiltrative and destructive. The presence of ORM1 in OM possibly can justify the classical mucoid appearance of this tumor. However, in the immunohistochemical assays we only observed a cytoplasmic expression of this protein, whereas extracellular expression was not detected.

## Conclusions

Our results showed that protein profiles of OM and DF are very similar, supporting the hypothesis that OM could originate from DF. We also identified eight proteins with differential expression between these samples. By Western blot and immunochemistry we confirmed the overexpression of the ORM1 protein in OM. This protein was located in the cytoplasm of stellate and spindle-shaped cells of OM as well as in the endothelial cells of large and small blood vessels. The properties and functions of ORM1 in this tumor are not clear, although the current evidence suggests possible immunomodulatory and/or angiogenic properties of this glycoprotein in the biological behavior of OM.

## Methods

### Tissue samples

Tissue samples were provided from the Department of Maxillofacial Surgery of the Juarez Hospital in Mexico City. The protocol was approved by the institutional committee of research and ethics under the registration number HJM 1996/11.03.08. For proteomics and Western blot analysis a total of five cases of OM diagnosed during the period between 2009 and 2011 were used in this work (Table1), as well as five samples of DF, the latter used as control tissues. Besides to the samples previously described, for immunolocalization assays of ORM1 we added nine OM and five DF samples, previously fixed in 10% neutral formalin and paraffin-embedded, which were obtained from the service of Maxillofacial Surgery of the Hospital Juarez de México and from Universidad de la República (UDELAR) Uruguay.

### Tissue preparation

DF were separated from the mineralized tooth or extracted from alveolar bone in the routine extraction of the third molars from 16–20 year-old people. Then, samples were cleaned using physiological solution, introduced to liquid nitrogen and stored at −70°C until use. OM specimens were removed during surgery, cleaned with physiological solution, frozen in liquid nitrogen and stored at −70°C. In addition, paraffin-embedded sections of eleven OM and five DF samples were examined by immunohistochemical assays.

### Protein extraction

Protein extraction of DF and OM was based on the selective extraction method described by Gorg et al. [54] and Perez et al. [55] with minor modifications. Briefly, samples were rinsed in physiological solution, frozen in liquid nitrogen, mechanically pulverized and suspended (400 mg tissue/ml) in sample buffer (7 M urea, 2 M tiourea, 4% CHAPS, 2% IPG buffer, 40 mM DTT) containing complete™ protease inhibitor cocktail (Roche, Germany). Then, samples were disrupted by sonication. Insoluble material was removed by centrifugation (20,000 xg for 5 min at 4°C), and the supernatant was preserved. Additionally, proteins were precipitated with acetone-TCA and the 2D Clean-Up Kit (Amersham Biosciences, USA). The precipitate was diluted in rehydration solution (7 M urea, 2 M thiourea, 2% CHAPS, 0.5%, IPG buffer and 0.1% bromophenol blue) supplemented with 2 mM DTT. Protein concentration was measured using 2D Quant Kit (Amersham Biosciences, USA) according to the manufacturer’s recommendations.

### Two dimensional electrophoresis (2-DE)

Protein extracts suspended in rehydration solution (250 μl) were used to rehydrate Immobiline Drystrip Gels, pH 3–10 of 13 cm (GE Healthcare, USA) for 18 h at room temperature. Electrofocusing was performed in an Ettan IPGphor 3 Isoelectric Focusing System (GE Healthcare, USA) at 16–20 kVh for 5 h. Then, the immobilized pH gradient (IPG) strips were incubated for 10 min in reducing and alkylating 2-DE equilibration buffer (6 M urea, 75 mM Tris–HCl, pH 8.8, 29.3% glycerol, 2% sodium dodecyl sulfate and 0.1% bromophenol blue) plus 65 mM DTT and 135 mM iodoacetamide, successively. For SDS-polyacrylamide gel electrophoresis (SDS-PAGE), a standard vertical electrophoresis system was used with 10% polyacrylamyde gels (15 cm × 13 cm) in a Gibco BRL V16 gel system. Gels were stained with Colloidal Coomassie Blue G-250 (Bio-Safe Coomassie Stain, Bio-Rad Laboratories, USA). A digital image of the gels was obtained using scanning densitometry (Image Scanner, Amersham Biosciences, USA) and analyzed with Image Master 2D Platinum software, version 7.0 (GE Healthcare Life Sciences, Switzerland).

### Identification of overexpressed proteins in OM

Six spots consistently overexpressed and two underexpressed in OM were excised, subjected to in-gel tryptic digestion and analyzed by LC-MS/MS [56]. Peptide mass fingerprinting and MS/MS data were searched against the human genome database using the MASCOT 2.1 program (http://www.matrixscience,com) allowing a monoisotopic mass tolerance of 1 Da. Methionine oxidation and one missed tryptic cleavage were used during the database search.

### Western blot

Western blot assays were performed as previously described [57]. Briefly, proteins in rehydration solution were separated by 10% SDS-PAGE and transferred to nitrocellulose membranes. After blocking for 2 h in 5% nonfat milk in Tris-buffered saline (TBS) containing 0.05% Tween-20, membranes were incubated with a monoclonal antibody against the orosomucoid 1 protein (ORM1) (1:5000) (Abcam, UK) and then with an anti-mouse secondary antibody conjugated to horseradish peroxidase (Invitrogen, USA) (1:10,000). As internal control, samples were probed with antibodies against α-actin (kindly provided by Dr. Manuel Hernández-Hernández, CINVESTAV-IPN). Antibody detection was developed by chemioluminescence (ECL, GE Healthcare Life Sciences, Switzerland). Relative intensities were documented and analyzed by densitometry.

### Histopathology and Immunohistochemical staining

OM and DF specimens were fixed in 10% buffered formalin and embedded in paraffin. Tissue sections of 2 μm thick were obtained and stained with hematoxylin and eosin, under standard procedures. All slides were reviewed for histopathological classification of odontogenic tumors according to the recent classification of head and neck tumors of the World Health Organization [1].

For immunohistochemical studies, tissue sections from fourteen OM and ten DF samples were treated with 0.1 M sodium citrate (pH 6.2) and Tween-20 for the unraveling of the epitopes. Endogenous peroxidases were blocked with 0.9% hydrogen peroxide, followed by incubation with 1% BSA in PBS for 5 min, in order to eliminate non-specific binding. Then, samples were incubated with monoclonal antibodies against ORM1 (1:200), and then with a biotinylated anti-mouse antibody, and finally with the streptavidine/peroxidase complex (LSAB + Labeled streptavid-Biotin, Dako Corporation, USA). The reaction products were visualized by incubation with 3,3´-diaminobenzidine-H_2_O_2_ as substrate (Dako Corporation, USA). Sections were counterstained with Mayer´s hematoxylin solution and visualized by optical microscopy. As a negative control, PBS was applied to substitute the primary antibody.

## Abbreviations

2-DE: Two-dimensional electrophoresis; DF: Dental follicle; IPG: Immobilized pH gradients; LC-MS/MS: Liquid chromatography-tandem mass spectrometry; OM: Odontogenic myxoma; ORM1: Orosomucoid 1 protein; SDS-PAGE: SDS-polyacrylamide gel electrophoresis.

## Competing interests

The authors declare that they have no competing interests.

## Authors’ contributions

AGM conceived and carried out experiments, analyzed data and drafted the manuscript; MAR conceived and designed the study, analyzed data and drafted the manuscript; RBM participated in the design of the study, analyzed data and helped to draft the manuscript; FECR and FCHH participated in the design of the study and analyzed data; JEFM carried out the immunohistochemical assays; GMH performed the mass spectrometry analysis; JJT and CLE were involved in collecting samples for this study and clinical data analysis. All authors read and approved the final manuscript.
